# Huggable integrated socially assistive robots: exploring the potential and challenges for sustainable use in long-term care contexts

**DOI:** 10.3389/frobt.2025.1646353

**Published:** 2025-10-15

**Authors:** B. M. Hofstede, S. Ipakchian Askari, T. R. C. van Hoesel, R. H. Cuijpers, L. P. de Witte, W. A. IJsselsteijn, H. H. Nap

**Affiliations:** 1 Vilans, Centre of Expertise for Long-Term Care, Utrecht, Netherlands; 2 Human-Technology Interaction group, Department of Industrial Engineering and Innovation Sciences, Eindhoven University of Technology, Eindhoven, Netherlands; 3 The Hague University of Applied Sciences, The Hague, Netherlands

**Keywords:** huggable integrated socially assistive robots, HI-SARs, social robots, daytime structure support, activity monitoring, social companionship, long-term care, older adults

## Abstract

With ageing populations and decreasing numbers of care personnel, care technologies such as socially assistive robots offer innovative solutions for healthcare workers and older adults, supporting ageing in place. Among others, SARs are used for both daytime structure support and social companionship, particularly benefiting people with dementia by providing structure in earlier stages of the disease and comfort in later stages. This research introduces the concept of Huggable Integrated SARs (HI-SAR): a novel subtype of SARs combining a soft, comforting, huggable form with integrated socially assistive functionalities, such as verbal prompts for daytime structure, interactive companionship, and activity monitoring via sensor data, enabling the possibility of more context-aware interaction. While HI-SARs have shown promise in Asian care contexts, real-world application and potential in diverse long-term care contexts remain limited and underexplored. This research investigates the potential of HI-SARs in Dutch healthcare settings (eldercare, disability care, and rehabilitation) through three studies conducted between September 2023 and December 2024. Study I examined HI-SAR functions and integration in Dutch care practice via focus groups with professionals, innovation managers, and older adults (N = 36). Study II explored user preferences through sessions with clients with intellectual disabilities and professionals (N = 32). Study III involved two case studies in care settings with clients and caregivers (N = 4). Results indicate that HI-SARs were generally well-received by professionals and older adults, who appreciated their support for daily routines and social engagement, particularly for clients with cognitive disabilities such as dementia. However, concerns were raised about hygiene, the functioning of activity monitoring, and limited interactivity. Based on these findings, we recommend four design and implementation strategies to improve the effectiveness of HI-SARs: (1) integrating personalisation options such as customizable voices to increase user acceptance; (2) optimising activity monitoring by simplifying data output and using sensor input more proactively to trigger interactions; (3) considering persons with cognitive impairments as a first target user group; and (4) encouraging individual use to enhance hygiene and tailor experiences to client needs. Overall, this research demonstrates the potential of HI-SARs in diverse long-term care settings, although further research is needed to explore their applicability, usability, and long-term impact.

## 1 Introduction

Due to double-ageing societies and the growing need for independent living among older adults, there is an increasing focus on care technologies within long-term care to support healthcare personnel in their daily jobs and older adults ageing in place (Runde, 2022). Among others, daytime structure and social engagement can be considered important factors to support older adults in ageing in place. Social engagement is important because a lack of it (i.e., social isolation and loneliness) has been linked to cognitive decline (Cardona and Andrés, 2023; Fowler et al., 2024; Kok et al., 2024b), wellbeing, emotional health and physical health (Koszalinski et al., 2025). Likewise, daytime structure plays an important role because it has been associated with several positive health outcomes, such as improved physical function (O’Conor et al., 2018).

A variety of care technologies have been developed to support ageing in place (Ienca et al., 2017). These include assistive care robots, which can provide physical, social, or medical assistance (Bouwhuis, 2016). Focusing on socially assistive robots (SARs), these robots either add a social component to physical or medical assistive robots, or they solely assist people through social interaction (Abdi et al., 2018). Additionally, Bouwhuis (2016) describes four robotic types of SARs: assistive (e.g., daytime structure support), companion (e.g., social presence), talking (e.g., verbal interaction), and emotional (e.g., through a huggable or comforting form). Besides, Vercelli et al. (2018) classify SARs as providing assistance by acting as caregivers, providing reminders and instructions for daily life, supporting in monitoring health and behaviour, or providing companionship. Hence, they describe clear overlap with Bouwhuis’ categories, along with additional complementary assistive functions related to monitoring. Furthermore, Ipakchian Askari et al. (2024) found that the social functionalities of SARs seem particularly useful in care for older adults and people with cognitive impairments.

Some SARs are primarily designed to support daytime structure by providing reminders for activities such as meals, medication, and appointments. An example is a stationary tabletop robot, shaped like a flowerpot, that delivers verbal reminders and engages in social interaction to encourage adherence to daily routines (Søraa et al., 2020; Søraa et al., 2021). Other examples include digital or virtual companion applications, which similarly provide reminders and social interaction (Bults et al., 2024; ConnectedCare, 2022). In contrast, robotic pets such as the widely studied therapeutic cuddly seal called ‘PARO’ address emotional support and the companionship domains (Shibata and Wada, 2011) of the different SAR types by Bouwhuis. Although PARO does not use speech, it responds to touch and provides older adults with social and emotional benefits by encouraging interaction and reducing stress (Hung et al., 2019).

Most SARs to date operate within one or two of Bouwhuis’ functional SAR types. However, recent developments show a trend toward integrating SARs with other domains. For example, in the European eWare project by Casaccia et al. (2019), where a SAR was connected with a life pattern monitoring system, which can also be one of the assistive functions of SARs as outlined by Vercelli et al. (2018). This allowed the SAR to provide more context aware and accurately timed reminders based on sensor data from the life pattern monitoring. Furthermore, the integration enabled tracking whether the reminders of the SAR were followed up on, for example, by checking if there was kitchen activity after the SAR reminded participants to have a meal. Casaccia et al. (2019) found that this integration resulted in better-aligned advice and more accurate measurement of activities of daily living (ADL) by older adults.

Another recent innovation comes from South Korea (Lee et al., 2019), where a robotic doll was designed to integrate three of Bouwhuis’ functional robotic types: it provides daytime structure (assistive), engages in speech-based interaction (talking/companion), and offers comfort through a huggable form (emotional). Furthermore, the robot collects sensor data from various points, including buttons in its hands and ears, a microphone, and a touch sensor on the forehead. Additionally, some versions contain a presence sensor in the neck. These sensors enable the monitoring of basic user activity patterns (e.g., detecting if a person is present in the room or holding the robot), a function we refer to as activity monitoring. However, achieving deeper insights and truly context-aware interactions goes beyond these basic reactive responses and requires advanced AI (Nap, 2024; Shen et al., 2025). AI can significantly increase the accuracy of monitoring and potential interventions (Jovanovic, et al., 2022; Kok et al., 2024a). For example, AI could enable the robot to learn user behavioural patterns from sensor data, allowing it to react proactively, such as greeting a user at their usual waking time. As AI technology advances, its possibilities become infinite, for example, the robot could even process speech to recognize signs of stress or cognitive decline in end-users (Ding et al., 2024; Haque and Deb, 2022).

Despite this clear potential of AI to further improve this type of robots, the current level of AI integration for such complex functionalities is still in development within these robots, and therefore, not the focus of this paper. Instead, this research explores user experiences of these robots in diverse care contexts as will be elaborated on later. Furthermore, given the unique combination of their huggable physical form and integrated functional characteristics (daytime structure support, social companionship, activity monitoring), a specific description for this novel type of robots seems appropriate. Therefore, we introduce the term:

**Huggable integrated socially assistive robots (HI-SARs)** These are SARs that combine a physically comforting (huggable) form with integrated socially assistive functions, such as emotional support, verbal interaction (talking/companion), daytime structure support (assistance), and activity monitoring.

The HI-SAR development can be interpreted in two ways: either as enriching non-cuddly SARs with emotionally engaging, huggable designs; or as enhancing cuddly SARs with proactive features like activity monitoring and daytime structure support. These two interpretations highlight the added value of integrating multiple functionalities into a single system. Moreover, such integrations enable HI-SARs to provide consistent and adaptable support across various stages of dementia. SARs offering structured prompts are especially helpful in the early to middle stages of dementia, when users can still follow routines (Lee et al., 2020; Ross and Rodriguez, 2021). Conversely, emotional SARs like robotic pets tend to be more effective in later stages, when the need for comfort and companionship becomes more prominent (Takayanagi et al., 2014; Carbone et al., 2022; Ipakchian Askari et al., 2024). By combining these complementary strengths, HI-SARs offer a more continuous and holistic form of support throughout the entire course of the dementia progress, thereby increasing their applicability across different phases of cognitive decline.

Following positive results on the use of the HI-SAR developed in South Korea, various research has been conducted to confirm its effectiveness in South-Korea and Japan (Lee et al., 2019; Lee et al., 2023a; Lee et al., 2023b). While the HI-SAR has demonstrated positive outcomes in Asian care contexts, it is interesting to examine whether this integrated SAR can be as effective in diverse care settings. Cultural differences and the unique needs of older adults in different care contexts may influence the effectiveness of such technologies (Broadbent et al., 2009). These differences vary in terms of aesthetic design preferences, as well as cultural aspects influencing user-acceptance and trust, or the preferred functionalities and communication norms. For example, the use of anime aesthetics in robot design is more prevalent in Asian cultures than in non-Asian ones (Li et al., 2010). Furthermore, Li et al. (2010), found that individuals from low-context cultures, such as Germany, showed significantly lower engagement with robots during less social tasks, compared to individuals from high-context cultures like China and Korea. In addition, Shibata (2012) noted that PARO is perceived differently across cultures ranging from a clinical tool to a robotic pet, highlighting the role of cultural acceptance across studies conducted in Europe, US and Asia. These findings suggest that cultural factors may influence how HI-SARs are received outside of Asia. Hence, these findings highlight the importance of testing whether HI-SARs can deliver comparable benefits in diverse cultural and diverse care settings, and therefore, we focus on a Dutch care context in this research.

While the HI-SAR concept is relatively new, two concrete implementations have emerged to date: the one developed in South Korea and a more recent one in the Netherlands. The latter shares notable similarities in both design and functionality with its South Korean predecessor. Given these overlaps, they can arguably be considered comparable HI-SARs. This makes the Dutch case particularly valuable for examining whether such integrated huggable assistive robots, originally designed and tested in Asian contexts, can also be meaningfully implemented in diverse long-term care environments.

Although HI-SARs have not yet been implemented in care contexts outside of Asia, elements of their functionality are already being used in Dutch care context. Technologies supporting social companionship, daytime structure, and activity monitoring are each present in various forms: care robots that provide verbal prompts and reminders, robotic animals that foster emotional engagement, therapeutic robotic dolls used with children with autism (Huijnen et al., 2016; van Hoesel et al., 2024), weighted stuffed dolls used for comfort in eldercare (Sumioka et al., 2021; Chang-Kredl et al., 2024; Krystyniak, 2020; Field, 2010), and life pattern monitoring systems that track daily routines (Morresi et al., 2024). However, the integration of these elements into a single intelligent and huggable robotic system is a novel development in the Netherlands.

To examine the applicability of HI-SARs in Dutch long-term care settings, we conducted three studies. The first study aimed to assess potential user groups and applications of HI-SARs in the Netherlands. The second study explored its use among older adults in residential disability care, focusing on desired features such as voice interaction and gender preferences. The third study involved deploying a HI-SAR in a rehabilitation care setting and in two departments (psychogeriatrics and somatics) of an eldercare organization. Together, these three studies aimed to explore the potential of HI-SARs in Dutch long-term care by integrating social interaction, daytime structure support, and activity monitoring. Through this research, we aimed to answer the following question: What are the potentials and challenges of utilizing Huggable Integrated Socially Assistive Robots (HI-SARs) in Dutch long-term care, and for which user groups do they provide added value?

## 2 Methods

This research comprises three studies conducted between September 2023 and December 2024 by researchers from Vilans, the national centre of expertise on long-term care in the Netherlands, in collaboration with the Hague University of Applied Sciences and Eindhoven University of Technology. The studies aimed to explore the potential of HI-SARs in long-term care, using a combination of semi-structured interviews, focus groups, and pilot testing.

### 2.1 Participants

Participants for Study I and II were recruited through four collaborating care organizations that responded to a LinkedIn invitation for participation. Additionally, two organizations participated as pilot test sites for Study III, as they were already engaged in testing the HI-SAR in coordination with the developer. Across these studies, participants included healthcare professionals, informal caregivers, and clients from eldercare, disability care, and rehabilitation care. To ensure privacy and reduce participant burden, only their roles (e.g., healthcare professional or client) were recorded. The sample sizes varied per study, with the numbers shown in Table 1. In total, 45 healthcare professionals, 2 innovation managers, 20 older adults, 1 rehabilitation client and 1 informal caregiver participated in the three studies.

**TABLE 1:** Number of participants per study.

Study	Number of participants
Study I: exploring the possible application of HI-SARs in Dutch long-term care	19 healthcare professionals, 15 older adults, 2 innovation managers
Study II: exploring client and caregiver preferences of HI-SARs	5 older adults, 24 healthcare professionals
Study III: two case studies with a Dutch HI-SAR (rehabilitation care and eldercare)	2 healthcare professionals, 1 informal caregiver, 1 rehabilitation client

### 2.2 Materials

**FIGURE 1:**
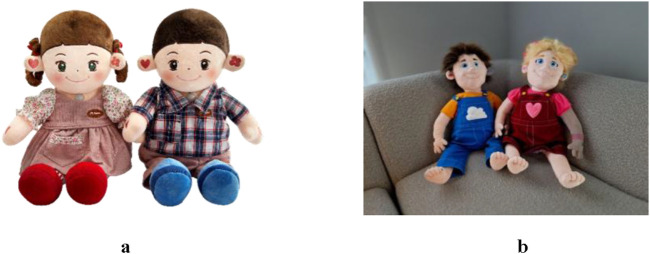
The Hyodol **(a)** and Maatje-pop **(b)** that have been used as HI-SAR in this research. Retrieved 6 January 2025, from Hyodol (n.d.) and SmartRobot.Solutions (n.d.).

Two HI-SARs were used across the three studies. For Study I, the Hyodol^1^ robot was used, and for Study II and III the Maatje-pop^2^ was used. These robots offer similar functionalities but have slightly different appearances, as can be seen in Figure 1. Both were presented with variations in appearance (e.g., male or female) and the Maatje-pop also in variations of voice (e.g., child-like or adolescent). Furthermore, both HI-SARs contain a set of sensors: a touch sensor in the head, pressure sensors in both ears and hands, and a microphone. Additionally, they contain an audio speaker and a vibrating device to simulate a heartbeat. All three studies involved interview guides which are available in Dutch upon request from the author.

### 2.3 Data-analysis

All three studies utilized qualitative methods, including interviews or focus groups, to gather in-depth insights into the potential of HI-SARs in Dutch long-term care. During the three studies notes were made, which served as input for the data analysis. In order to protect privacy, no audio recordings or transcripts were made. In Study III, the data was analyzed by means of open coding, to identify concepts and patterns in the data in a structured manner.

For Studies I and II, a thematic analysis approach, as described by Clarke and Braun (2018), was employed to explore patterns and themes within the data. Thematic analyses were conducted in two iterative stages. In the first iteration, one author (BH) systematically reviewed and categorized all notes into broad thematic clusters, such as appearance and functionalities. These broad categories helped to organize the data for further analysis. In the second iteration, two authors (BH and SI) collaboratively reviewed, refined, and discussed the themes. This process involved identifying overarching themes and subthemes, ensuring that they accurately captured the perspectives of participants. In case of disagreement during this analysis process, the other authors were involved in the discussion to reach consensus.

### 2.4 Ethical considerations

Informed consent was obtained from all participants, and participating care organizations provided approval. The research was partly conducted by Vilans, a knowledge organization in long-term care that ensures adherence to ethical standards, including consultation with a privacy officer as part of its internal ethical procedures. Potential risks, including privacy concerns and emotional wellbeing, were carefully assessed to ensure responsible research practices.

### 2.5 Procedures

#### 2.5.1 Study I: exploring the possible application of HI-SARs in Dutch long-term care

This study, conducted between September 2023 and December 2023, explored the potential of a HI-SAR (Hyodol) in the Netherlands, aiming to hypothesize its value for long-term care and determine how it could be optimally integrated into the Dutch care context.

The study began with desk research to create an overview of the functionalities of the HI-SAR and how these compare to other care robots. Then, workshops were held with eight healthcare professionals to map the process of providing daytime structure support and identify opportunities for the integration of a HI-SAR in such a process. Using care process mapping techniques, participants outlined existing workflows and discussed potential roles for the robot.

To further explore end-user perspectives, four focus groups were conducted. Two of those sessions involved healthcare professionals, and the other two involved older adults. The focus groups focused on first impressions, expectations, practical considerations, and ethical concerns related to HI-SAR implementation. All four focus groups followed a semi-structured format, beginning with an introduction to the HI-SAR and its functionalities, followed by asking for informed consent and guided discussions on the interview topics.

In the final phase, an effect mapping session was conducted by two researchers (BH and TH), in which the output of the earlier conducted focus groups were used to create an overview of the hypothesized effects of using a HI-SAR. The hypothesized effects were then structured into an effect map, available in Dutch in the Supplementary Material.

#### 2.5.2 Study II: exploring client and caregiver preferences of HI-SARs

This study took place from February 2024 to June 2024 and focused on exploring client and caregiver preferences for the functionalities, voice, and perceived gender of HI-SARs. The research activities aimed to gain insight into the factors influencing acceptance and user experience. Initially, a focus group was conducted with five older adults with intellectual disabilities and two supporting caregivers to gather their impressions of interacting with Maatje-pop, which was used as a HI-SAR in this study. This was followed by semi-structured interviews with three participating clients from the focus group to delve deeper into their preferences and perceptions, using a Wizard-of-Oz method (Kelley, 1984) to explore different voice variants employing both a child’s recorded voice and an adult voice generated via text-to-speech technology as described by van der Bij (2024). Focus group involving 22 healthcare professionals was held to reflect the findings. Participants also discussed potential applications and interaction opportunities for HI-SARs in Dutch healthcare contexts.

#### 2.5.3 Study III: two case studies with Dutch HI-SAR (rehabilitation care and eldercare)

**FIGURE 2:**
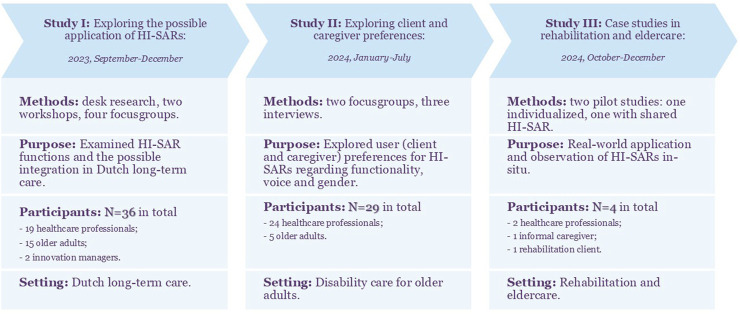
Conceptual overview of the three HI-SAR studies: methods, purpose, participants, and setting. Created by the authors.

The pilot study took place between October and December 2024 and explored the usability and impact of a HI-SAR in real-world care settings. It was conducted in collaboration with two care organizations that served as pilot sites for the developer of Maatje-pop. At a rehabilitation care facility, a client recovering from a stroke used the HI-SAR for 1 week, and two interviews were conducted: one with the client and their informal caregiver and one with a formal caregiver. At an eldercare organization, the robot was tested over 4 weeks across somatic and psychogeriatric departments, with one healthcare professional providing insights based on observations and experiences with approximately forty clients. The study aimed to assess the HI-SAR’s practicality in Dutch long-term care, perceived benefits, and areas for improvement. An overview of all study-specific methods and procedures is provided in Figure 2.

## 3 Results

### 3.1 Study I: HI-SAR exploring the possible application of HI-SARs in Dutch long-term care

Study I started with gaining insights into the functionalities of HI-SARs and comparing it to other care robots and weighted stuffed dolls. This comparison is shown in Table 2.

**TABLE 2:** Comparison of different socially assistive tools.

	Cognitive support and daytime structure	Activity and social stimulation		Comfort and emotional support			Integrated social assistance
Example(s)	Tinybots Tessa^3^ 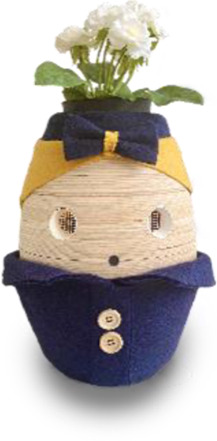	Maatje-robot^4^ 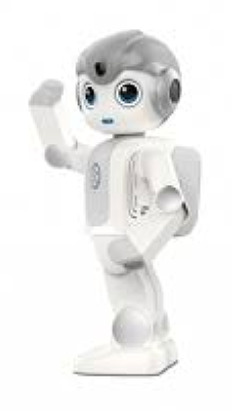	Zora^5^ 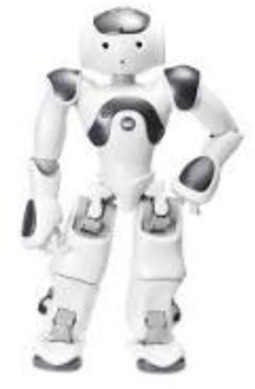	Paro^6^ 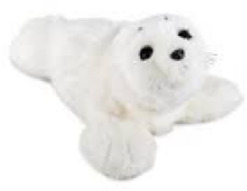	HUG^7^ 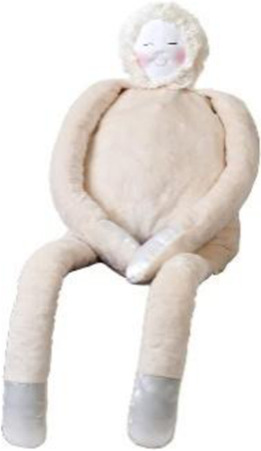	Weighted stuffed dolls N/A	HI-SARs (Hyodol^8^, Maatje-pop^9^) 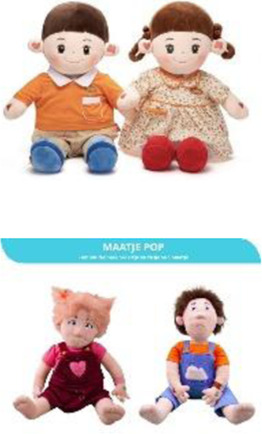
User group	People with cognitive disabilities (dementia, autism, or another psychosocial condition)	People with dementia, autism, and mild intellectual disabilities	Not for a specific target group. Primarily used with older adults and other people in care	People with mild cognitive impairments or dementia	People with mild cognitive impairments or dementia	People with mild cognitive impairments or dementia	People with mild cognitive impairments or dementia
Movements (actuators)	No movement	Movement of the limbs	Movement of the limbs	Movement of the body (seal)	Heartbeat simulation	No movement	No movement
Detection(sensors)	Audio (speech recognition), push button (1x)	Audio (speech recognition), camera (for scanning QR codes)	Audio, (infrared) camera, facial recognition, sonar, tactile and pressure sensors	No	No	No	Audio, motion and touch detection, push buttons in the hands and ears
Battery life	Not applicable (designed for continuous mains power)	Approximately 2–3 h on a full charge	Battery life not specified; Equipped with a 62.5 Wh lithium-ion battery (21.6 V)	Not specified, but designed for long-term use	2–3 days	Not applicable	3–7 days
Interaction	Speak written messages, eye-blinking	Speak written messages with a preset limb movement and emotional tone	In current applications not possible to send messages, but possible to respond to questions	Nonverbal, sensory-based interaction in terms of sounds and movements	Can simulate a heartbeat and play the sound of a heartbeat	No interaction	Speak written messages, play MP3 files of recorded messages, maintaining the voice of the speaker
Scripts	Can execute scripts. There are predefined scripts, but it is also possible to write a custom script	Can execute scripts. This script can be composed with, for example, appointments, words, songs, voice pitch, and speed	Zora is fully programmable and can answer questions	No scripts	No scripts	No scripts	Contains a complete script (which can be personalized regarding the client status)
Functions	Providing daytime structure support (agenda function), playing music, and asking (preset) questions to which the user can respond with yes or no.	Games, educational tools, physiotherapy and rehabilitation exercises, stories or responses based on interest, providing daytime structure support, responding to signaling plans	Facial recognition, stimulate movement, play music, dance, read stories, and play games	Provide comfort and serenity by cuddling	Provide comfort and stress relief by cuddling and simulating a low heartbeat by sound and resonance	Provide comfort and serenity by cuddling	Provides daytime structure support, plays music and quizzes, religious stories, provide comfort and serenity by cuddling, and movement exercises

Based on the focus group transcriptions of Study I, six themes (Appearance, Hygiene, Functions, Target group, Individual use, and Sensor data) and four subthemes were found by the thematic analysis. The themes and subthemes are discussed in the following subsections.

#### 3.1.1 Theme 1 (Appearance): although most caregivers were positive about the appearance and material of the HI-SAR, some caregivers perceived it as childish or unrealistic

Most caregivers found the appearance of Hyodol appealing, describing it as cute or friendly, and ascribed this to factors like the colours and the softness of Hyodol. They appreciated that Hyodol resembled a child without making it too realistic. However, some caregivers and one of the participating older adults felt the design was unrealistic or too childish, which could make it less suitable for certain care settings in which clients live without advanced cognitive decline, such as somatic care units. Others noted that it might appeal more to women than men.

##### 3.1.1.1 Subtheme 1a (Weight): caregivers were positive about the weight of the HI-SAR

In general, caregivers appreciated the weight of Hyodol, stating that it contributed to a sense of presence. Some caregivers indicated that the weight HI-SAR made it realistic as a child, rather than feeling like a lightweight toy. While the majority of the caregivers expected that the current weight of Hyodol was manageable for older adults, some caregivers mentioned that a slightly heavier version could enhance this effect even more.

##### 3.1.1.2 Subtheme 1b (Material): the softness of the HI-SAR was received positively by caregivers and older adults

Hyodol’s softness was well-received among participants (i.e., caregivers as well as older adults), with caregivers highlighting the comforting texture of Hyodol, particularly for bedridden clients. Observations during the focus group with older adults showed that clients instinctively hugged Hyodol, reinforcing its suggested comforting nature. However, caregivers noted that the hard electrical component on the back reduced its overall huggability.

#### 3.1.2 Theme 2 (Hygiene): caregivers raised questions about cleaning and hygiene

Hygiene was a key concern among caregivers. They questioned whether Hyodol could be easily cleaned or washed and suggested that protective treatments, such as materials permeated with antimicrobial agents, might improve hygiene. Nevertheless, they still expressed concerns regarding hygiene and asked whether the technology developer had thought about this. Observations during the focus group with older adults confirmed these concerns are not unfounded, as clients often held Hyodol close to their mouths and sometimes drooled on it, emphasizing the need for proper cleaning methods and individual ownership.

#### 3.1.3 Theme 3 (Functions): caregivers see the HI-SAR as valuable to activate older adults through daytime structure support and music, and to comfort them by music, touch, and social companionship

Caregivers expect that Hyodol can remind individuals of daily activities, which can activate the older adults and also reduce repetitive questions asked by clients, which eases the burden on caregivers. Furthermore, caregivers also expect Hyodol can serve as a social companion, engaging users in small conversations or prompting them to recall past experiences, which can provide comfort but is also expected to activate the clients in engaging in social interactions. The activating role was confirmed during the focus group with older adults where of the participants expressed willing to dance together with Hyodol, or to listen to music together or to recall past experiences.

*“We can’t be present 24/7, but Hyodol helps keep residents engaged, active, and reassured throughout the day.” - Caregiver*

Apart from the activating role music can have, Hyodol was especially appreciated for its calming effect, aiding in relaxation during care moments or reducing anxiety. Additionally, Hyodol’s tactile nature was expected to encourage interaction, particularly for individuals with advanced dementia who rely more on sensory input than verbal communication.

While caregivers acknowledged that Hyodol cannot replace human care, they saw it as a valuable tool to complement their work, offering structure, stimulation, and emotional support.

##### 3.1.3.1 Subtheme 3a (Dementia journey): the HI-SAR can be used throughout the complete dementia journey

Caregivers and older adults highlighted the different functions of Hyodol suitable for different stages of cognitive decline in clients. For example, they noted that daytime structure and verbal messages that require language processing by the end-user are more suited for clients with higher cognitive abilities, while comforting and calming are expected to better meet the needs of clients with lower cognitive abilities. One of the caregivers summarized this by asking to one of the older adults: ‘Do you want it as a little child or as an assistive tool?’.

Caregivers see potential for Hyodol to assist older adults in the first stages of dementia in maintaining independence by providing reminders for daily activities, medication, and appointments. As dementia progresses, Hyodol shifts towards offering emotional support and companionship, becoming a familiar presence that provides comfort. Furthermore, the ability to personalize interactions based on user needs is expected to make Hyodol a flexible tool that evolves alongside the individual, ensuring continuous support throughout their dementia journey.

*“At first, it helps with structure, but later, it becomes more of a companion. It can grow with the disease.” - Caregiver*

##### 3.1.3.2 Subtheme 3b (Stress relief): the HI-SAR could relieve distress among clients through its companionship and huggable appearance

The soft and huggable design of Hyodol was appreciated by caregivers, who expect that many clients will instinctively embrace it. Consequently, Hyodol is expected to provide a sense of comfort, particularly for clients experiencing agitation or loneliness. The older adults expressed liking Hyodol as well and indicated they would like to use it for comfort and to combat loneliness. The relaxing effects of music, touch, and companionship were seen as important factors in reducing distress. The observation that most of the older adults immediately started hugging Hyodol reinforced these expectations. Therefore, the ability of Hyodol to engage through touch and sound makes it a potentially valuable tool in calming clients, offering continuous emotional support.

#### 3.1.4 Theme 4 (Target group): caregivers see people with cognitive disabilities as the target user group for HI-SARs

Caregivers see people with dementia (PwD), mild cognitive impairment (MCI), and other cognitive disabilities as the primary target group for HI-SARs because these individuals might benefit from structured support and social companionship. Hyodol is expected to help maintain daily routines, reduce anxiety, and provide a reassuring presence, the latter two especially as cognitive decline progresses. In contrast, caregivers expect that individuals without cognitive impairments may find Hyodol unnecessary, and those with primarily physical needs may require more practical assistive devices. Additionally, some clients rejected Hyodol during the focus group, a notion which was expected by some caregivers as they ascribed it to stigma, viewing Hyodol as childlike and expecting it to be unnatural for some clients to interact with a doll. As a result, Hyodol is seen as most valuable for clients experiencing cognitive decline who are expected to experience less stigma.

*“It’s really suited for people in the early stages of dementia, but also for those with cognitive disabilities who might benefit from companionship and structure.” – Caregiver*

*“I have played enough with dolls already when I was a child.” – Older adult*

#### 3.1.5 Theme 5 (Individual use): for effective use, the HI-SAR should be deployed individually and be personalized

The tailoring of Hyodol to meet the individual needs of each client was mentioned as important by caregivers. Personalisation, including preferred music, familiar phrases, and relevant reminders, is expected to enhance engagement. Additionally, caregivers noted that although using Hyodol in a group setting is possible, the effectiveness is expected to be the highest in individual settings. Moreover, caregivers expect a sense of ownership by the clients, which can play an important role in the usability and acceptance of Hyodol. This expectation of ownership was confirmed during the focus group with older adults, at which some of the participants did not want to return Hyodol after hugging with it.

*“Each client has different needs. The HI-SAR should be set up specifically for them—otherwise, it won’t have the same impact.” - Caregiver*

#### 3.1.6 Theme 6 (Sensor data): data collected by the sensors can be used by FC and IC to monitor the usage of the HI-SAR and the emotional status of clients

Caregivers saw potential in the ability of Hyodol to collect data through sensors, which could help formal caregivers (FC) and informal caregivers (IC) to monitor engagement and probably even emotional wellbeing. By tracking usage patterns, movement, and responses, caregivers could gain insights into the mood of clients and potential changes in their behaviour.

*“If we can see patterns in how often someone interacts with Hyodol, it might help us detect changes in wellbeing before they become bigger issues.” - Caregiver*

However, some of the caregivers also acknowledged concerns about data overload, with, on the one hand, privacy concerns related to the data collection and, on the other hand, limited time for healthcare professionals to gain actionable insights from all these data. Nevertheless, the caregivers still expressed seeing value in using these insights, for example, to provide more responsive and personalized care.

### 3.2 Study II: exploring client and caregiver preferences of HI-SARs

Based on the focus group transcriptions of Study II, four themes (Positive response, Initiated interaction, Functions, and Voice) and one subtheme (Gender) was found by the thematic analysis. The themes and subtheme are discussed in the following subsections.

#### 3.2.1 Theme 1 (Positive response): clients and care professionals responded positively to the HI-SAR, appreciating its appearance, clothing, and hair

Clients were generally enthusiastic about the look of Maatje-pop, noting its clothing and hairstyle as appealing. Some participants also expressed interest in dressing the robot themselves, for example, changing its clothes based on the season.

*“I really like it. I like the clothes.” – Older adult*

There even was curiosity about whether the robots were real, indicating an engagement with their lifelike qualities, as one of the older adults repeatedly asked whether Maatje-pop was real or not.

*“But those are not real? Not real?” – Older adult*

##### 3.2.1.1 Subtheme 1a (Gender): both the boy and girl versions of the HI-SAR were well-received by clients and perceived as suitable for providing comfort

Both the male and female versions of Maatje-pop were appreciated by the older adults and both seen as capable of providing emotional support. While there were varying preferences among the older adults, which version of Maatje-pop they liked the most, overall, there was no clear preference for one over the other, with opinions being roughly split. Most older adults indicated that Maatje-pop could be used for providing comfort.

#### 3.2.2 Theme 2 (Initiated interaction): most clients initiated verbal interaction with the HI-SAR, but there was some confusion about physical or verbal interaction initiated by the HI-SAR

Many clients naturally engaged in verbal interaction with Maatje-pop, showing interest in communicating with it. Talking to the robot seemed to be an intuitive and enjoyable experience for most participants. Fewer clients engaged in physical interaction, such as hugging or petting the robot. Furthermore, when Maatje-pop initiated physical interaction, reactions among older adults varied. Some participants responded immediately and eagerly hugged the robot, holding onto it for a prolonged period. Others, however, did not respond to the verbal request of Maatje-pop for physical interaction. These differing responses indicate that some clients naturally embrace the idea of physical contact with Maatje-pop, while others remained unresponsive.

The initiated verbal interactions by Maatje-pop also caused confusion in some situations. Especially in the cognitive games where an ambiguous question (e.g., what colour is a rose?) was asked by Maatje-pop and participants did not know how to respond.

*“I do not understand what I should do.”- Older adult*

#### 3.2.3 Theme 3 (Functions): clients value the HI-SAR for its companionship as well as for its activation through games, conversations, and daytime structure support

Maatje-pop was valued not only as a source of companionship but also as a tool for activation and social engagement. Clients would like using it in various contexts, such as playing games, having conversations, walking, singing together, and receiving comfort. This suggests that there are two clear ways in which clients would like to engage with Maatje-pop: some saw it as a companion, enjoying its presence for comfort and emotional support, others saw it as an activation tool, using it for cognitive stimulation and maintaining daily routines.

*“I would like it to comfort me when someone is ill.” – Older adult*

*“I would like to do a puzzle with it in the afternoon.” – Older adult*

The heartbeat function of Maatje-pop was discussed as well among the clients and while some found it reassuring and helpful for sleeping, others either found it too intense or did not fully understand its purpose. This suggests that while features like the heartbeat function can enhance the comforting role of Maatje-pop, they may need adjustments or better explanations.

*“It [the heartbeat function] goes fast.” – Older adult*

#### 3.2.4 Theme 4 (Voice): clients have a preference for a HI-SAR with an adult voice that is gender-congruent

There was a general preference among older adults for Maatje-pop to have an adult voice that matched its gender, as the child-like voice was sometimes harder to understand. During one-on-one interactions, some clients explicitly mentioned that they found the adult voice easier to hear and comprehend. However, one of the participants found both voice options appealing and did not have a strong preference. This indicates that while an adult voice may be the more universally accessible option, some flexibility in voice options could accommodate a broader range of user preferences.

#### 3.2.5 Focus group

The results regarding the intended usage of HI-SARs and its corresponding functionalities throughout the day varied. For the morning, most participants indicated that the robot could be used as an agenda or alarm clock. Additionally, some mentioned cuddling and singing together. Other possible activities and interactions mentioned by the participating healthcare professionals included comforting, eating together, distraction during caregiving, watching TV, hugging, weather updates, news, and talking. For the afternoon, social activities such as singing together, playing games, cuddling, and dancing together were most frequently mentioned. Some participants also suggested activities such as talking, reading together, giving kisses, hugging, meditating, eating together, watching TV, comforting, and being close. For the evening, all caregivers stated that Maatje-pop could be used for comforting, and some mentioned using it for activities like sleeping together, reading together, and cuddling. A few also suggested using the robot for time-checking, dancing together, talking, giving kisses, playing games, and watching TV. Two healthcare professionals did however express concerns that proactive HI-SARs would take over their healthcare-related tasks that they experience as enjoyable.

When asked what tone of voice would be most suitable for the target group (older adults with cognitive impairments), caregivers responded as shown in Table 3. The results show that both the child’s voice (score of 7 for the female and 8 for the male) and the adult voice (score of 7 for the female and 6 for the male) were preferred for both the female and male robot. The robotic voice received the lowest score (score of 1 for both the female and male dolls). This partly corresponds with the preferences of the clients, who showed a stronger preference for an adult voice due to its clarity.

**TABLE 3:** voice preferences for both versions of Maatje-pop (female vs. male).

	Female version	Male version
Voice of a child	7	8
Voice of an adult	7	6
Robotic voice	1	1

### 3.3 Study III: two case studies

The three semi-structured interviews were initially analysed by the first author (BH), with participants’ responses clustered according to the predefined interview topics. Subsequently, corresponding clusters were combined and for each of these clusters the first author derived overarching themes (Support and Engagement, First Impressions, Functionalities, Dashboard, Unmet Expectations, and Expected Effects). The results are presented below, organised according to these overarching themes.

#### 3.3.1 Theme 1 (Support and engagement): the HI-SAR was expected to support and engage clients

In both care settings, caregivers expressed that Maatje-pop was expected to support clients when caregivers are not directly available. Additionally, in rehabilitation care, the expectations were that the robot could provide comfort and improve the daily structure of the clients with reminders and music. The informal caregiver, who was the husband of the rehabilitation client, expected the robot to engage his wife more without overburdening the formal caregivers and himself. In psychogeriatric care for older adults, it was expected that the robot would be effective due to its human-like characteristics.

#### 3.3.2 Theme 2 (First impressions): positive first impressions and initial effects that diminished over time in rehabilitation care, with client-specific potential in care for older adults

In both care contexts, interviewees’ initial impressions of Maatje-pop were generally positive. In care for older adults, the robot was well received according to the interviewed caregiver due to its appealing design, user-friendly operation, and the familiarity of its operating system (similar to Robot-Maatje). The intuitive interface was appreciated, and in some cases, Maatje-pop offered comfort, particularly for clients experiencing loneliness or restlessness. Some clients embraced the robot and seemed to view it as a companion. However, according to the interviewed caregiver, acceptance was client-specific, as not all older adults were open to interacting with a doll. Moreover, effects such as reduced agitation have not yet been scientifically confirmed and require further research according to the interviewed caregiver.

In rehabilitation care, both the informal and formal caregiver were initially enthusiastic. They valued the ease of use and the robot’s potential to activate clients. Over time, however, this enthusiasm declined. The robot’s activating potential was more limited than expected, mainly due to repetitive responses (e.g., consistently saying “nice that you pet my head”). This lack of variation sometimes led to boredom or even agitation in the client, who at times perceived Maatje-pop as childish. There was also confusion by the formal caregiver caused by differences between the functionality discussed during the instruction session and what was actually available in the demo version.

While Maatje-pop was initially received positively across settings, these findings indicate that its longer-term effectiveness may be limited in rehabilitation care. However, according to the formal caregiver in care for older adults, the robot still shows promise, especially when tailored to individual client needs and their willingness to engage.

#### 3.3.3 Theme 3 (Functionalities): although the music function was received positively in both care settings, some other functionalities caused confusion or overstimulation

Experiences with Maatje-pop’s functionalities varied between rehabilitation care and care for older adults, though some commonalities emerged as well. Music was positively received in both settings: it brought enjoyment and relaxation in rehabilitation care and activated some clients in care for older adults. However, in rehabilitation care, the client sometimes became overstimulated by Maatje-pop and put it away when caregivers were not present. Music and therapy reminders were well received in the rehabilitation care setting, though there was a desire for more variety in the demo version.

In care for older adults, the robot worked well for clients with early-stage dementia, while some functionalities, such as the quiz and news or weather reports, caused confusion or were too challenging for clients with more advanced dementia. Music, the quiz, and daytime structure reminders were the most frequently used features in care for older adults. While the quiz provided engagement for some, it was often either too simple or too difficult, depending on the client. Daytime structure reminders proved effective, and interactive elements like responding to petting helped create a calming presence.

Some functionalities, such as the weather report and games, were found to be too complex or less relevant, particularly in rehabilitation care. Thus, while Maatje-pop showed promise, its features had to be carefully matched to the abilities and needs of individual clients to avoid overstimulation or confusion.

#### 3.3.4 Theme 4 (Dashboard): caregivers perceived the dashboard as user-friendly

The dashboard and controls were user-friendly. Caregivers in both settings found it easy to adjust settings, thanks to the clear buttons and intuitive interface. They noticed a lack of variety in music in the demo version. Additionally, there was a request to add custom voice options and to set automatic daily schedules–two features that are already possible in the new version but were not in de demo version that the participants tested with.

The informal caregiver in rehabilitation care wanted more interactive functions, such as personalized language options tailored to the user’s cognitive level and socio-economic background.

#### 3.3.5 Theme 5 (Unmet expectations): although the HI-SAR has not yet met the expectations of the informal caregiver in rehabilitation care, its potential is clearly recognized by him

The informal caregiver in rehabilitation care found the music feature of Maatje-pop insufficient, as the robot offered only four standard songs during the pilot. According to the informal caregiver, this limited selection contributed to the client’s boredom, even when interacting with Maatje-pop, and made the robot less effective in practice. Despite this, the informal caregiver recognized the potential of Maatje-pop, especially in terms of entertainment and activation. He mentioned that the robot could help entertain the client, giving him time for other tasks and potentially reducing caregiver strain.

However, neither the informal nor the formal caregiver uploaded additional songs to better match the client’s personal preferences, although the technology developer indicated this was possible. This suggests how essential personalisation is for the success of features like music. The pilot showed that certain preferences, such as music, are client-specific, and, for example, incorporating an intake list of favourite music genres could enhance the robot’s ability to meet individual needs. While the current implementation fell short, the informal caregiver still indicated to see future possibilities for Maatje-pop if such personalisation and interactivity are improved.

#### 3.3.6 Theme 6 (expected effects): while no clear effects were experienced by healthcare professionals in rehabilitation care, the HI-SAR did help reduce workload in elderly care

In rehabilitation care, the healthcare professional found it difficult to determine whether the use of the demo version of Maatje-pop affected their workload. Healthcare professionals had to set up the schedule daily and actively offer Maatje-pop to the client, as the client did not use it independently.

According to the interviewed caregiver in care for older adults, Maatje-pop could potentially reduce the workload, especially if it reduces restlessness in clients, as “caregivers literally have their hands free”. However, this depends on how caregivers use Maatje-pop. The caregiver explained that if the added value of Maatje-pop is not seen or its functions are not used to their fullest potential, it is often put aside, so better staff training and motivation are crucial.

## 4 Discussion

This research comprised three studies that collectively provide a number of insights to improve the potential and reduce challenges associated when implementing a huggable integrated socially assistive robot (HI-SAR) in diverse care contexts. After discussing our results, we will offer four recommendations: two on HI-SAR design and two on HI-SAR implementation in long-term care.

### 4.1 The added value of integrating three technological applications in one robot

Our findings suggest that the HI-SAR’s integration of various applications can enhance its relevance and adaptability in different care contexts. Caregivers across our studies valued the HI-SAR not only for its capacity to provide social companionship and support daytime structure, but also indicated to see potential to respond more dynamically to client needs. For example, combining sensor data with activation functions could allow the HI-SAR to prompt interaction when client engagement is low, supporting more personalized and proactive interventions. Similarly, emotional input could be used to trigger comforting features such as music or tactile feedback. This reflects findings by Casaccia et al. (2019) and Nap et al. (2024), who showed that combining multiple assistive technologies can improve care outcomes by reinforcing each other’s functionality and tailoring interventions more precisely to user needs. In line with Casaccia et al. (2019), who demonstrated how the combination of a SAR and lifestyle monitoring tools improved the timing and relevance of reminders, our results suggest that such integration can enrich care interactions by making them more context aware. Likewise, Nap et al. (2024) emphasized how interconnected assistive technologies can strengthen decision-making algorithms and better align with the dynamic needs of people with dementia. Together, these studies support the idea that HI-SARs, which integrate multiple technologies, can offer more adaptive and effective support in care settings, especially when integrated with AI models (Shen et al., 2025).

The notion that a layered approach (i.e., integrating care technologies) is expected to enhance effectiveness aligns with literature highlighting the importance of personalized interventions in care robotics. For instance, Kaptein et al. (2015) emphasize that tailoring robot-assisted care to individual needs is key to its success and that a layered approach facilitates this by enabling the combination of various technologies and functionalities, allowing for more precise customization of support. Furthermore, Tapus et al. (2009) emphasize that adaptive robot behaviour, based on real-time monitoring, can significantly improve user engagement and emotional wellbeing. Likewise, Leite et al. (2013) found that SARs that adapt to user states are more likely to be perceived as empathetic and supportive, which can boost acceptance and long-term interaction. Hence, the integration of multiple technologies can have positive outcomes related to the effectiveness of healthcare technologies, as well as related to user engagement and acceptance, and the emotional wellbeing of end-users.

However, our studies also reveal the complexity of implementing such integration. In the focus groups of Study I, caregivers expressed concern about data overload from activity monitoring of the HI-SAR, questioning whether they had the time or capacity to interpret the information meaningfully. This reflects broader concerns in the literature about data fatigue and the risk of overwhelming caregivers in already demanding environments (Lukkien et al., 2023). Furthermore, the case studies on rehabilitation care and eldercare of Study III showed that while the monitoring feature was present, it was not actively used in the care routines by the formal and informal caregivers, limiting its perceived value.

Although not all functionalities, such as the activity monitoring feature, were actively used by (in)formal caregivers, this does not necessarily indicate a shortcoming of HI-SARs. Rather, it reflects the flexibility of integrated systems, allowing caregivers to tailor usage to the individual needs and contexts of clients and seems in line with the balancing act of responsible innovation, as argued by Lukkien D. et al. (2024). For some, monitoring may offer added value by enabling subtle observations of mood and activity; for others, companionship or music may be more relevant. This flexibility in function aligns with the required adaptability within the framework of Care Centered Value Sensitive Design in healthcare, as outlined by van Wynsberghe (2016), who emphasizes the importance of developing technology that can respond to the dynamic needs of both caregivers and clients. While simpler, minimalistic solutions may sometimes suffice (Sumioka et al., 2021), our results indicate that integrated systems like the HI-SAR could also be of value, because the integrated functions can complement or strengthen each other and making it possible for the technology to be used in the early stages of dementia and adapt as the disease progresses to more severe stages of dementia.

### 4.2 The potential of HI-SARs in the Netherlands

The three studies demonstrated the potential of HI-SARs in Dutch care settings, which may also hold relevance in other diverse care contexts. Across all three studies, the majority of both clients and caregivers responded positively to the HI-SAR, especially appreciating its softness, appearance, clothing, and hair; findings that resonate with research emphasizing the importance of robot aesthetics and material type in user acceptance (Broadbent et al., 2009). Furthermore, according to the literature review by Flandorfer (2012) older adults in various cultural contexts tend to prefer robots that are not overly human-like. This observation relates to the uncanny valley effect (Mori, 1970), which suggests that as the appearance of a robot becomes more human-like, the affinity of people toward it increases to a certain point. When the resemblance becomes almost but not fully human, people tend to experience discomfort or even aversion, known as the uncanny valley. The positive evaluations in our studies suggest that the HI-SARs successfully balance incorporating enough human traits to foster social engagement, without appearing too human-like. Its soft and doll-like appearance seems to avoid mimicking the human form too closely, allowing it to maintain a clear non-human identity while still being perceived as approachable and engaging. This balance may in part be due to the HI-SARs’ anime-inspired aesthetic. While such a design was initially expected to hinder acceptance in non-Asian settings due to cultural differences compared to Asia (Li et al., 2010), it may have actually contributed to the robot’s success. The stylized, non-realistic look of anime may have helped differentiate the HI-SAR from lifelike human representations, supporting social interaction while avoiding the discomfort associated with the uncanny valley effect.

Furthermore, Bartneck and Forlizzi (2004) argue that the appearance of robots can contribute to the construction of a sense of identity in interactions. This refers to the way users attribute personality, social roles, and emotional capacity to a robot based on its design features. Such sense of identity influences how users relate themselves to the robot and how they engage in interactions with the robot. In our studies, the HI-SAR’s design likely contributed to such sense of identity. Clients and caregivers highlighted elements such as the HI-SAR’s softness, clothing, and hairstyle as appealing. One older adult clearly expressed liking the HI-SAR and especially its clothes, while others expressed interest in changing its outfits. Caregivers and older adults described the HI-SAR as cute or friendly, appreciating that it resembled a child without being too realistic. The robot’s weight also played a role, with caregivers noting it felt more like holding a real child than a toy. Clients were observed hugging the robot, and some even questioned whether it was real, suggesting a strong sense of engagement. Therefore, design factors like softness, clothing, hair and colors, can probably form the sense of identity towards the HI-SAR and help clients in perceiving the HI-SAR not just as a tool, but as a social companion with a recognizable and comforting presence. Furthermore, the ability of the HI-SAR to be such a comforting presence and social companionship were found in the current studies and ascribed to contributing to the perceived potential of using a HI-SAR in the Netherlands. Additionally, the ability for activation was also found in our studies, contributing to the perceived potential of the HI-SAR.

In the focus groups of Study I, caregivers recognized the value of HI-SARs in activating older adults and providing them with comfort, whereas in the focus groups of Study II, clients particularly valued the HI-SAR for its companionship and its ability to activate them through games, conversations, and daytime structure support. The case studies in rehabilitation care and eldercare of Study III showed that especially the comforting role and companionship was available in the current versions of the HI-SARs. Furthermore, Study III revealed that music was well-received, bringing both enjoyment and activation to clients in different care settings. The effect of activating older adults with dementia by a robot that reads together and plays music was also researched by Cooke et al. (2010), which showed positive effects in terms of improved self-esteem and sense of belonging.

Additionally, the case study in eldercare suggested that the HI-SAR might be able to reduce the workload of caregivers. Thereby, providing them more time to attend to other tasks or for interaction with clients. This finding confirms the expectations as expressed in Study I and II, and also supports the broader literature on the potential of socially assistive robots (SARs) to reduce caregiver burden by taking over certain routine or socially supportive tasks (Feil-Seifer and Matarić, 2011; Gasteiger et al., 2023). As such, our case study adds to the growing body of evidence that SARs can act as complementary tools in care environments, supporting not only clients in their need for autonomy (Rogers and Mitzner, 2017) but also enhancing the workflow and efficiency of caregiving staff. Furthermore, the portability of the HI-SAR was received as useful as well, as became clear in the case study on eldercare. Portable and lightweight design is increasingly seen as an important factor for integrating social robots into real-world care environments (van Wynsberghe, 2016) and is also a factor that is appreciated by older adults, as was found by Flaldorfer (2012).

It was expected that the South-Korean HI-SAR would face scepticism in other cultures and thrive primarily in Asian cultures, where robots are more culturally embedded in nursing contexts (Broadbent et al., 2009) and the anime style is more present in everyday life. However, the results indicate that Dutch clients and caregivers are receptive to the technology, suggesting potential for broader acceptance in diverse care settings. Therefore, our findings demonstrate that HI-SARs have the potential to contribute in various ways to the quality of care in Dutch care institutions, benefiting both clients and caregivers.

### 4.3 Limitations

While the findings of the studies offer valuable insights, several limitations must be acknowledged. One limitation is that the findings of Study I have not been validated in real-world practice. Furthermore, the case studies of Study III had a small sample size, particularly in the rehabilitation setting, where only one client participated. As such, the results primarily offer expectations on the usage of HI-SARs, and further field studies with larger and more diverse samples are needed (Bryman, 2016). However, the insights gained from this study are still valuable, as they provide a glimpse into the potential of HI-SARs and can guide more targeted evaluations in future studies to confirm whether the hypothesized findings hold true in a broader context.

Another limitation of this exploratory research is the intentional omission of detailed demographic data beyond professional roles. This choice was made to prioritize participant privacy and align with the process of technology explorations. Consequently, factors such as education level, technology propensity, or prior experience with robots, which could offer deeper insights, were not investigated, and hence, form a suggestion for future research.

Additionally, the HI-SAR was tested in a demo version with some functionalities disabled or being unclear due to the absence of training programs for healthcare personnel. The limited or inappropriate use of certain functionalities may have influenced caregiver and client perceptions. However, despite the possible frustration caused by this limited or inappropriate use of the early prototype, user feedback remains crucial for refining the design at each stage, and thus, early design phases as well (Lim et al., 2006). While we acknowledge this as a limitation, we recognize it as an inherent challenge when striving to involve users in all phases of the design process (Suijkerbuijk et al., 2019).

Furthermore, there is a potential participant bias, particularly among caregivers. The care organizations involved were those that responded positively to a LinkedIn call, which may have excluded the more sceptical care organizations. This could have led to a positive bias in the findings (Robinson, 2014). However, the effect can be diminished because feedback from multiple caregivers within each organization was collected, and therefore, this research still offers valuable insights and a solid foundation for future studies.

Another limitation is the potential novelty effect (Reimann et al., 2024), particularly in Studies I and II and, to a lesser extent, in Study III. The introduction of a new product like the HI-SAR may generate initial positive reactions simply due to its novelty. Over time, enthusiasm may wane among end-users, as seen in Study III, where enthusiasm declined during the rehabilitation care case study. Though based on a single case, this suggests the novelty effect may influence user experiences. Future research should conduct longer-term pilot studies with the recommended user groups to assess whether observed effects are sustainable or driven by initial novelty.

Cultural factors may also play a role, and while the HI-SAR was well-received in the Netherlands, its applicability in diverse contexts remains uncertain. Previous studies have shown cultural differences in acceptance of robotic technology (Nomura, 2017; Broadbent et al., 2009). Finally, concerns from caregivers about task replacement need to be addressed through co-design processes that include caregivers in the development and implementation phases to ensure successful integration (Sanders and Stappers, 2008).

## 5 Recommendations for HI-SAR design and implementation

Based on the findings from our studies, we propose four recommendations to guide the further development and deployment of the HI-SAR in care settings. The first two recommendations are focused on the design of HI-SARs, the latter two are about the deployment in Dutch care context for older adults.

### 5.1 It is recommended to integrate personalized voice, appearance and functionality to increase user acceptance and effectiveness (Recommendation on design)

We suggest considering the personalisation of the HI-SAR’s features to increase its effectiveness and acceptance among a wider range of users. Clients in Study II showed a preference for a HI-SAR with a gender-congruent adult voice, with some finding the adult-like voice easier to understand compared to the child-like voice. This suggests that incorporating customizable voice options (e.g., different gender or age) can make the HI-SAR more accessible and effective (Gasteiger et al., 2023).

Additionally, personalizing the appearance of the HI-SAR (e.g., offering different clothing styles) could make the HI-SAR more relatable to clients, as caregivers and clients in Studies I and II reported they would like to dress up the HI-SARs. This can probably contribute to the sense of identity as well (Bartneck and Forlizzi, 2004), making the HI-SAR more suitable for social engagement. Moreover, it is important to personalize certain functionalities, such as the heartbeat function and cognitive games, which some clients found either too intense or difficult to understand. This aligns with recommendations from previous research (Leite et al., 2013), which highlighted the role of adaptive features in enhancing user engagement.

### 5.2 Focus on optimising the activity monitoring in the HI-SAR design: prevent data-overload and proactively use the data in the other HI-SAR functionalities (Recommendation on design)

Study I highlighted that caregivers have limited time to analyze the activity monitoring data, and therefore, the data should be presented as simply as possible and prevent data-overload. This finding was confirmed in Study III where the caregivers did not make use of the activity monitoring function. As Lukkien et al. (2023) discussed, the risk of overwhelming caregivers with excessive data must be mitigated. Therefore, we recommend further optimising this functionality.

Furthermore, Studies I and II suggest that integrating sensor data more proactively into the HI-SAR’s activities could strengthen its utility. For instance, if the HI-SAR detects limited usage, it could prompt a caregiver or trigger specific interventions to activate the client, such as starting a game or initiating a conversation. Similarly, monitoring emotional status could be used to activate comforting features when a client appears stressed or anxious, ensuring that the HI-SAR continues to provide both companionship and emotional support throughout the day. These insights resonate with Casaccia et al. (2019) and Nap et al. (2024), who demonstrated how combining assistive technologies improves the timing and relevance of interventions. Finally, these data insights should also be integrated into the digital-hybrid care processes, enabling proactive use for early detection and informed decision-making by caregivers.

### 5.3 When deploying a HI-SAR it is recommended to consider persons with cognitive impairments as a main target user group (Recommendation on deployment)

While our studies showed a positive reception of HI-SARs, it is important to consider the specific needs of the target population when deploying the technology. Our findings suggest that HI-SARs are particularly beneficial in settings involving individuals with cognitive impairments, such as people with dementia (PwD), mild cognitive impairments (MCI), or other cognitive disabilities. In Study I, caregivers specifically highlighted the added value of the HI-SAR for these groups, emphasizing its role in providing daytime structure support, cognitive stimulation, and emotional companionship. Findings that resonate with earlier work of Vercelli et al. (2018), Cooke et al. (2010) and Tapus et al. (2009). Conversely, for individuals without cognitive impairments or those with primarily physical care needs, the HI-SAR may be perceived as less relevant or even childish, as was observed in both case studies (Study III).

### 5.4 It is recommended that each user has their own individual HI-SAR and hygiene management is considered (Recommendation on deployment)

Finally, we recommend individualized usage among persons with cognitive impairments. Given that the HI-SAR’s effectiveness is influenced by the individual needs and preferences of clients, it is crucial to consider individual use when deploying the HI-SAR. This recommendation is in line with the suggestion to design HI-SARs with sufficient personalisation options (Recommendation 1) a principle consistently highlighted in the literature as key to maximizing the benefits of care robotics (e.g., Kaptein et al., 2015; Leite et al., 2013). Allowing each user their own HI-SAR can facilitate personalization and adaptation of the robot’s functionalities to the end-user’s unique and evolving needs, leading to enhanced engagement and perceived effectiveness. Moreover, the recommendation on individual usage is reinforced by the expressed concerns among caregivers about hygiene, as these concerns are less prominent with individual use of the HI-SARs. Furthermore, practical considerations such as hygiene management seem crucial for sustainable deployment. Our findings highlight caregiver concerns on hygienics, which could be addressed by developers exploring solutions like materials permeated with antimicrobial agents for easier cleaning or making dedicated washing services more universally available for HI-SARs.

## 6 Conclusion

This research introduced the concept of Huggable Integrated Socially Assistive Robots (HI-SARs) in Dutch care context and examined their potential for implementation in long-term care settings. While HI-SARs have been positively received in Asian settings, cultural and contextual differences raised questions about their universal acceptance and effectiveness. Nevertheless, our three interconnected studies suggest that HI-SARs are generally well-received by both caregivers and clients, particularly among individuals with cognitive impairments such as dementia or mild cognitive decline, indicating promising potential for implementation in diverse long-term care contexts. Their soft appearance, customizable features, and capacity to provide comfort, as well as stimulation and daytime structure support contribute to their acceptance.

At the same time, our findings suggest that personalisation (e.g., adaptive functionalities or speech characteristics) and improvement of the activity monitoring (e.g., prevent data overload, and use real-time sensor feedback) would be great assets in the design of HI-SARs to fully reach their potential. Furthermore, challenges such as novelty effects and limited real-world testing illustrate the need for further research and long-term evaluation of HI-SARs. For future research, we suggest to include economic evaluations such as a societal business case, gather more extensive participant data (e.g., education, prior technology experience), and explicitly address long-term ethical implications, including emotional attachment (Law et al., 2022) and more responsible innovation principles (Lukkien D. R. M. et al., 2024).

Taken together, the results suggest that HI-SARs can also enrich non-Asian care environments when thoughtfully designed and deployed. While not a one-size-fits-all solution, the flexibility of the HI-SAR offers promise in supporting both emotional wellbeing and caregiving workflows, particularly in cognitively focused care. Continued research and iterative development, in close collaboration with end-users, is suggested to ensure HI-SARs become a sustainable and meaningful addition to long-term care. Hence, by exploring this novel integration of huggable integrated SARs in Dutch care context, these findings provide a basis for further research and refinement on the universal use of HI-SARs.

## Data Availability

The raw data supporting the conclusions of this article will be made available by the authors, without undue reservation.

## Footnotes

1 Hyodol. (n.d.). Hyodol robot for elderly care. Retrieved 6 January 2025, from https://hyodol.oopy.io/

2 SmartRobot.Solutions. (n.d.). Maatje-pop: Zorgrobot voor ouderen. Retrieved 6 January 2025, from https://smartrobot.solutions/zorg-robots/maatje-pop/

3
Tessa ondersteunt zelfregie, zelfstandig wonen en biedt zorg.

4
Smartrobot.solutions - Robot verkoop, verhuur en ontwikkeling

5
https://www.zorabots.nl/onze-robots/

6
PARO Therapeutic Robot

7
Home | HUG by LAUGH

8
HYODOL: AI Care Platform

9
Maatje Pop - Smartrobot.solutions
