# Supplementary material for: Huggable integrated socially assistive robots: exploring the potential and challenges for sustainable use in long-term care contexts
Source: Front Robot AI. 2025 Oct 15;12:1646353. doi: 10.3389/frobt.2025.1646353 (PMC12569430; doi:10.3389/frobt.2025.1646353)
Supplement: Supplementary file 1 [file Supplementaryfile1.docx]

# Supplementary Materials

## A: The various research activities in Study I

| Table 1: complete overview of participants in study I | | |
| --- | --- | --- |
| **Research activity** | **Care setting** | **Participants** |
| Mapping the care process | Eldercare (daytime activities) | Healthcare professionals (N=4):  *3 caregivers, 1 case manager dementia* |
| Mapping the care process | Eldercare (intramural) | Healthcare professionals (N=4):  *3 caregivers, 1 therapist* |
| Focus group with healthcare professionals | Eldercare (intramural and extramural) | Healthcare professionals (N=4): *4 caregivers* |
| Focus group with healthcare professionals | Disability care (intramural care for individuals with severe intellectual disabilities) | Healthcare professionals (N=3):  *3 caregivers*  Innovation manager (N=1) |
| Focus group with older adults | Disability care (intramural care for individuals with severe intellectual disabilities and dementia) | Older adults (N=6) Healthcare professional (N=1):  *1 caregiver* |
| Focus group with older adults | Disability care (intramural care for individuals with mild intellectual disabilities and dementia) | Older adults (N=9) Healthcare professional (N=3):  3 *caregivers* Innovation manager (N=1) |

##
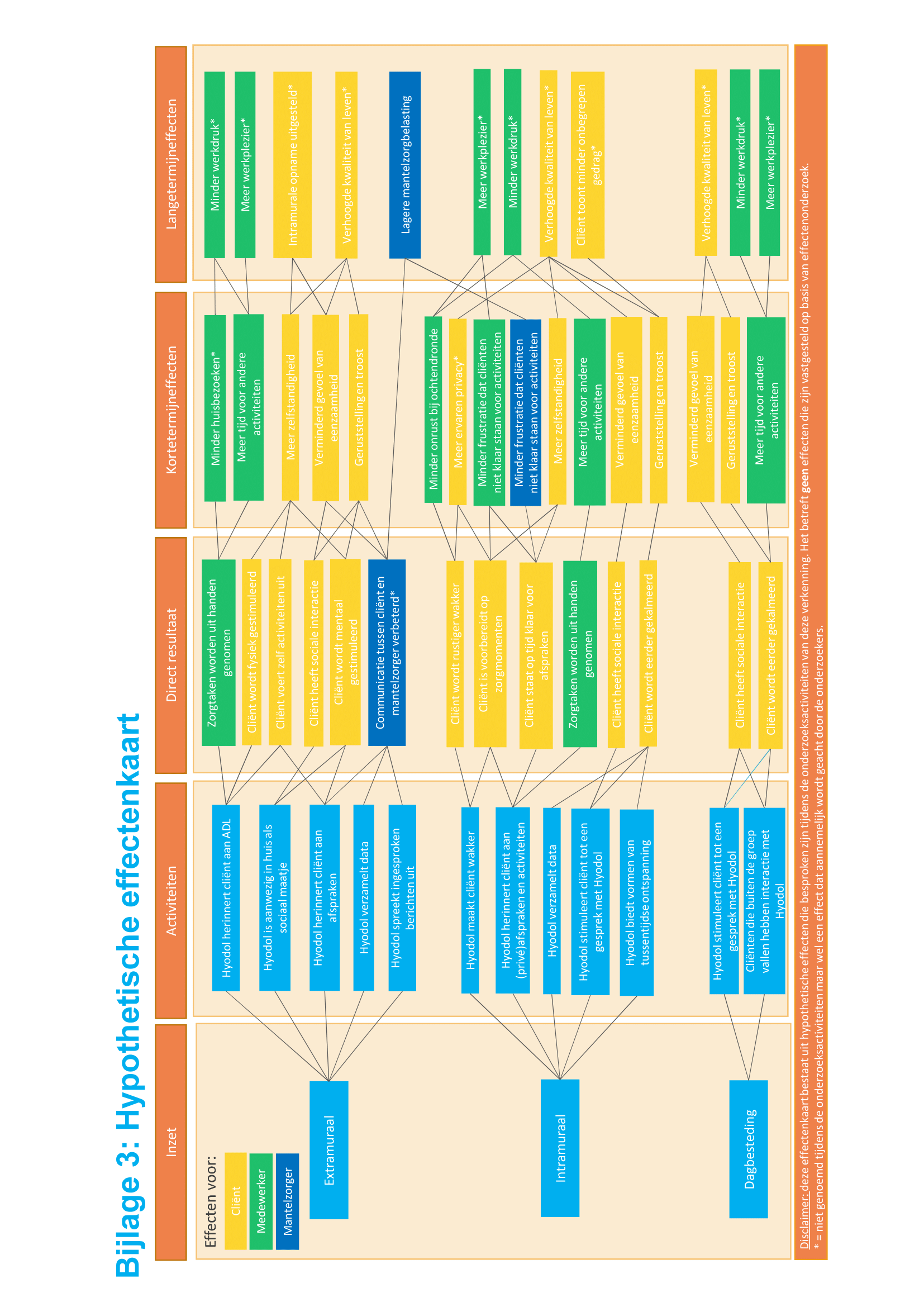
B: Hypothetical effect mapping based on Study I (only available in Dutch)
